# Use of reproducible research practices in public health: A survey of public health analysts

**DOI:** 10.1371/journal.pone.0202447

**Published:** 2018-09-12

**Authors:** Jenine K. Harris, Kimberly J. Johnson, Bobbi J. Carothers, Todd B. Combs, Douglas A. Luke, Xiaoyan Wang

**Affiliations:** 1 Brown School, Washington University in St. Louis, St. Louis, Missouri, United States of America; 2 Center for Public Health Systems Science, Brown School, Washington University in St. Louis, St. Louis, Missouri, United States of America; University of Newcastle, AUSTRALIA

## Abstract

**Objective:**

Use of reproducible research practices improves the quality of science and the speed of scientific development. We sought to understand use of reproducible research practices in public health and associated barriers and facilitators.

**Methods:**

In late 2017, we surveyed members of the American Public Health Association Applied Public Health Statistics section and others; 247 of 278 who screened eligible answered the survey, and 209 answered every applicable question. The survey included questions about file management, code annotation and documentation, reproducibility of analyses, and facilitators and barriers of using reproducible practices.

**Results:**

Just 14.4% of participants had shared code, data, or both. Many participants reported their data (33%) and code (43.2%) would be difficult for colleagues to find if they left their institution. Top reported barriers to using reproducible practices were data privacy (49.8%) and lack of time (41.7%). Participants suggested training (50.9%) and requirements by journals (44.4%) and funders (40.2%) to increase use of reproducible research practices.

**Conclusions:**

Increasing use of reproducible research practices is important for public health and requires action from researchers, training programs, funders, and journals.

## Introduction

Increasing rates of retraction of scientific research suggest that error, omission, and fraud threaten the quality of evidence we rely on to make policy and program decisions, including in public health [1–3]. For example, researchers trying to replicate studies were successful for just 21% of 67 drug studies [3], 40% to 60% of psychology studies [4], and 61% of economics studies [5]. Error and omission in reporting of statistical results are among the reasons for poor replication rates. In one sample of psychology papers, 6% of p-values were incorrectly reported [6] and 11% of p-values were incorrectly reported in a sample of medical papers [7]. In addition, 20 to 80% of papers in each of ten top scientific journals omitted or were unclear about sample sizes and up to 40% of papers in each journal did not include the type of statistical tests performed [8]. Another study found tables were mislabeled in three of six reproduced public health studies [9].

Poor quality research may have serious consequences for human health. More than 400,000 subjects were enrolled and 70,501 subjects were treated in studies later retracted [10]. Retracted papers continue to be cited frequently after retraction [11,12], presumably often in support of related research. Retraction Watch reports 500–600 scientific papers are retracted each year. Errors and other undisclosed reasons are accountable for 73.5% of retractions [2], while fraud is implicated in 26.6%.

The gold standard for confirming results and reducing error, omission, and fraud is *replication*, or conducting an entire study a second time to verify its results [13]. Because replication is not always feasible due to cost, reproducing a study, or analyzing an existing data source to produce the same study results, has been proposed as a minimum standard increasing the reliability of reported research findings. Research is *reproducible* when data are accessible and data management and analysis instructions are clear and complete [14–16]. Adopting reproducible research practices speeds up scientific discovery, fosters greater exchange of ideas among scientists, and reduces research waste [16,17]. In addition to these societal benefits, research papers with shared data have fewer errors, those with shared data or shared code are cited more, and shared data sources are the basis for more publications [17,18].

Even in light of the benefits of using reproducible practices, there remain concerns for scientists whose careers rely on scientific contribution. Concerns include the opportunity cost of spending time to prepare data and code for public dissemination, the chance that work will be more closely scrutinized, and the possibility that publishing opportunities will be preempted by another scientist [19,20]. These pressures not only limit adoption of reproducible practices, but also increase adoption of questionable practices that threaten reproducibility, including failing to report all dependent measures and excluding data based on results [21].

Policy and logistics can hinder adoption of reproducible practices as well. Restrictions from study sponsors or data sources are often a barrier for sharing of health data. For data without restrictions, it can be difficult to find a stable location for data and code, since supplementary information of published journals may be transient [22,23]. Finally, managing and maintaining shared data can be time consuming and data are often disorganized or even lost [24].

To date, most research on reproducibility has been in psychology and medicine. Our survey is among the first to examine current practices and challenges to the creation of a culture of reproducibility in public health. We sought to answer four questions: (1) To what extent are public health analysts and researchers sharing data and statistical code? (2) What are the barriers to sharing data and statistical code? (3) How are public health professionals organizing and storing data and statistical code? and (4) What are the perceived facilitators to increase reproducible research practices in public health?

## Methods

This project was funded by the Robert Wood Johnson Foundation (RWJF) Increasing Openness and Transparency in Research program. The survey was approved by the Institutional Review Board (IRB) at Washington University in St. Louis (Protocol ID #201703045).

### Participants

Given our focus on data management and analysis in public health, we selected the membership of the American Public Health Association Applied Public Health Statistics (APHS) section as the primary target for data collection. Emails inviting members to participate in the survey were sent to the 707 unique names found in the member directory at the time of data collection. We complied with privacy policies on the organization website. Links to the survey were also distributed on three Twitter feeds: @RWJF (Robert Wood Johnson Foundation), @JPHMPDirect (Journal of Public Health Management & Practice), and @coding2share. Before survey began, participants who had clicked on the link to the survey were shown a letter of consent and asked to choose one of the following: (1) I have read this consent letter and agree to participate, or (2) I do not wish to participate. For those who consented to participate, two screening questions at the beginning of the survey restricted participation to individuals who worked with quantitative data and had recently contributed to quantitative analyses in a published report or manuscript.

### Survey

The author team developed the survey using two resources: (1) interviews with researchers who work with public health data, and (2) existing published literature on research reproducibility, false research findings, code annotation, and style guidelines [3–7,10,16,20,25]. The interviews provided insight on working with large data sets, automated processes, version control, and team coding. The literature provided detail on coding practices and other aspects of research important to reproducibility. The survey can be found online at https://github.com/coding2share/Surveys.

At the beginning of the survey, participants were asked to identify a specific publication or report in the last year where they led or were involved in the statistical analysis. Participants provided a nickname for the publication or report; the nickname was used in many of the questions to serve as a specific example for their practices.

The survey focused on four main topics and included an open field at the end for any additional ideas related to reproducibility. *Data and code file management practices* included questions about how participants stored data and code. For example: “While working on [nickname], how did you store the data and statistical code used for analysis? (check all that apply): 1) In the cloud, 2) On a local server at your workplace, 3) On a desktop computer, 4) On a laptop computer, 5) on a removable storage device, 6) There were no statistical code files.” Parallel questions were often asked separately for data and statistical code.

*Code annotation and documentation* included questions about having a variable dictionary/codebook, what items they included in a prolog if they had one (e.g. project name), comprehensiveness of code comments, code development guidelines they may have followed (e.g. Google’s R Style Guide), and formatting practices (e.g. naming conventions).

*Reproducibility of statistical analyses* included questions about information made available with the publication and how the code was developed. Participants were asked whether they included the name of the software used, statistical approaches used, and sample sizes in the publication; if a clean version of the data, statistical code, project directory, and/or a readme file were made publicly available; if they were required to make data or code publicly available by a funder, journal, employer, or research team; and the level of teamwork used to develop the code (one person working alone, etc.).

*Research reproducibility facilitators and barriers* included questions asking about what would be most important to facilitate increased used of reproducible research practices (additional financial resources, training, etc.), barriers experienced (lack of time, data privacy, etc.), and what kind of format would like for reproducible research training (online live webinar, etc.).

The survey was conducted online using Qualtrics in late 2017. The survey was anonymous; no personal identification information was linked to the survey. We conducted descriptive analyses and developed figures in R [3.4.2]. The survey and the code and data used for analysis are available in the coding2share GitHub repository at https://github.com/coding2share/OpenSciSurveyPaper.

## Results

### Participant characteristics

A total of 247 of 278 who screened eligible filled out the survey, and 209 participants answered every applicable question. The mean age of survey participants was 40.1 (sd = 13.7) years old, and most (68.9%) were in Public Health, Biostatistics, or Epidemiology. More females (59%) than males (39.5%) took the survey, and just under half (43.5%) had a PhD or DSc. More participants used SAS most of the time (39.7%), than SPSS (19.2%), Stata (18.7%), or R (9.8%). A majority of participants were white (66.2%), 55.9% worked in academic settings, and just under half (%) had been in their current positions for four or more years.

### To what extent are public health analysts and researchers sharing data and statistical code?

Of 215 participants, a total of 31 (14.4%) shared code, data, or both in their recent manuscript or report. Specifically, 11 reported sharing data only, 6 shared code only, and 14 (6.5%) shared both data and code. Of the 195 who had not shared code we asked whether they had ever made statistical code publicly available; 22 (11.3%) answered that they had made code publicly available at some point.

We examined data and code sharing by academic job status and sex but found only minor differences in the proportions of participants sharing across these categories. Specifically, of 118 academics and 93 non-academics, 13 academics (11% of academics) and 12 non-academics (13% of non-academics) made data available after publication while 12 academics (10.2% of academics) and 8 non-academics (8.6% of non-academics) made code publicly available. Survey participants included 124 women and 83 men completed the survey. Of these, 15 women (12.1% of women) and 10 men (12% of men) made data available to the public after publication. Likewise 10 women (8.1% of women), 9 men (10.8% of men), and 1 participant missing a response to the sex question had made code publicly available after publication.

**Fig 1:**
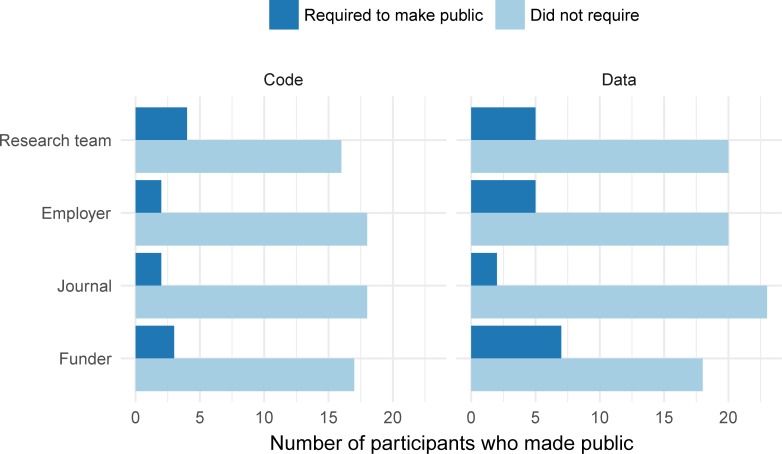
Of participants who made data (n = 25) or code (n = 20) publicly available, which (if any) entity required the data or code to be public? These responses are from the *reproducibility of statistical analyses* section of the survey.

For those who reported sharing data or code publicly, we asked whether the funder, journal, employer, or research team had required the data or code be public. Of the 25 sharing data, 15 (60%) were required by one or more of the four entities (i.e., funder, journal, employer, research team). Likewise, of the 20 sharing code, 7 (35%) were required by one or more of the four entities. Funders were most likely to require data be shared, while research teams were most likely to require code to be shared (Fig 1).

### What are the barriers to sharing data and statistical code?

**Fig 2:**
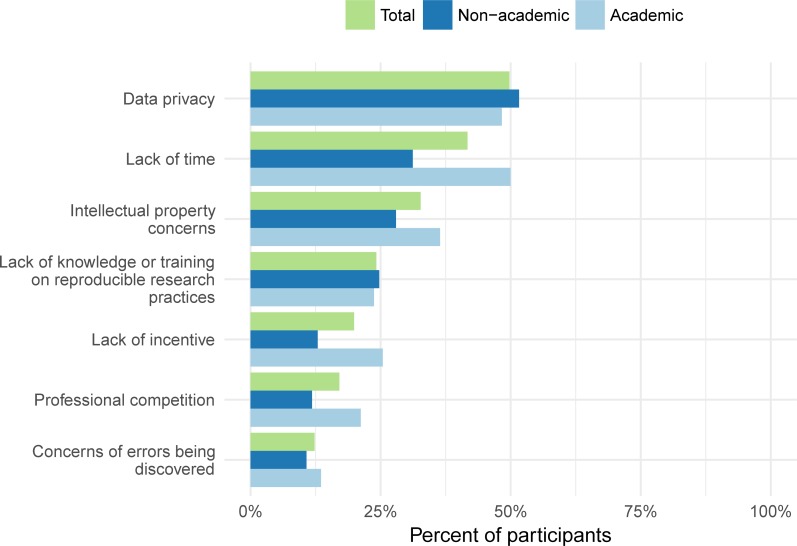
Percent of participants who perceived each of seven barriers to using reproducible research practices. These responses are from the *research reproducibility facilitators and barriers* section of the survey.

We asked participants to identify which of seven barriers to making data or code public they had experienced. We provided an open-ended *other* field and the option to choose *no barriers* or *have not tried to make data or statistical code available*. Data privacy was the most cited barrier with 105 participants (49.8%) checking this box. Lack of time and intellectual property concerns were the second and third most common barriers, while concerns about error discovery was chosen the least (Fig 2). Just 1 participant indicated no barriers, while 60 participants indicated they had never tried to make data or code available, and 9 participants wrote in responses including two citing organizational restrictions (“Restrictions on sharing by organization”), three citing external restrictions (“Funders own data, not us”), two logistics problems (“Datasets are too big”), and two citing lack of will or desire (“not sure it was desired by anyone”).

We anticipated barriers might be different in academic and non-academic settings. The top concern for academics was lack of time, followed closely by data privacy. Data privacy was the top response by far for non-academic participants. Academics also selected more barriers indicating they perceive more barriers than non-academics to making data and code public.

### How are public health professionals organizing and storing data and statistical code?

**Fig 3:**
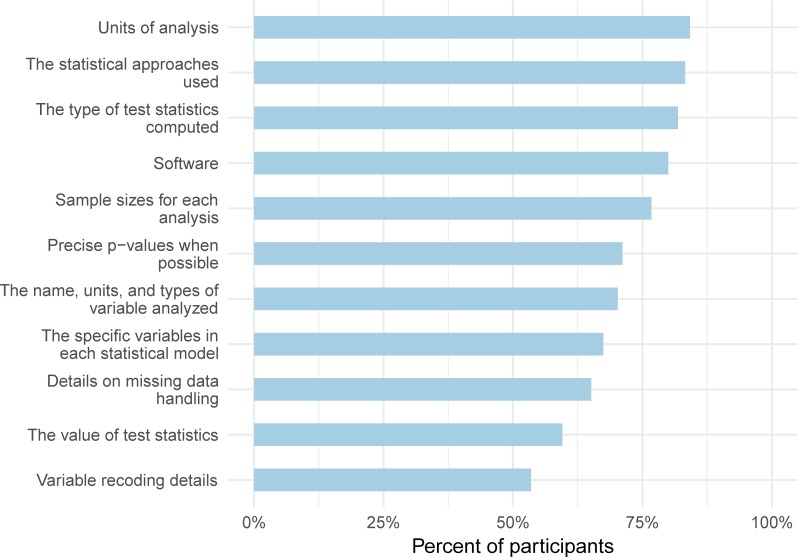
Percentage of participants who included each of 11 details that could facilitate reproducibility in recent publication or report. These responses are from the *reproducibility of statistical analyses* section of the survey.

While publicly available data and code are ideal for supporting reproducibility, including sufficient detail in publications and reports is also important, especially when code is not available or is available but not well-annotated. We asked participants whether their recent publication or report included each of 11 types of information about the data and analyses. While most participants included units of analysis and the statistical approaches used, under half included test statistic values or variable recoding details (Fig 3).

### Formatting and organizing data and code

Coding practices and file storage of data and code are important aspects of reproducibility. If files are not easily found and used, it may be difficult or impossible to reproduce analyses. We asked participants, “If you were to leave your institution suddenly, how easy would it be for someone to find the specific files for your recent publication or report without any instruction from you?” with response options on a four-point scale ranging from very easy to very difficult. A substantial number of respondents reported their data (33%) and code (43.2%) would be somewhat or very difficult for colleagues to find.

After finding the data and code, being able to understand the contents of the data and code files is essential for reproducibility. Understanding data files often requires a codebook listing the variables and how they were measured. Most participants (74.9%) reported having a codebook for their recent publication or report.

There are many promising practices and conventions recommended for writing clear code. Guidelines summarizing these practices are available for the major statistical software packages (e.g., SAS Style Guide). We asked participants if they followed any specific guidelines during statistical code development. The majority reported *not* following guidelines (64.1%) while 26.3% partially followed guidelines and 9.6% closely followed guidelines. When asked why they followed the guidelines, 50 of the 60 participants (83.3%) who followed guidelines reported that following guidelines makes their life easier, 41 said it produces better code, 38 reported being taught that way, 37 said it increases reproducibility, 28 thought it improves collaboration, and 11 and 6 said it was the policy of their research team or required for publication.

**Fig 4:**
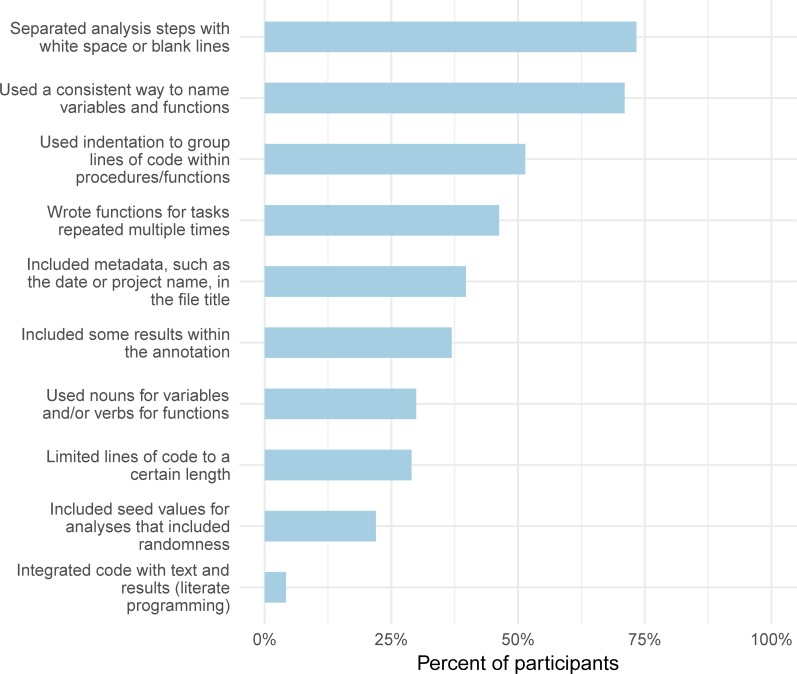
Percentage of participants who used coding practices recommended to facilitate reproducible research. These responses are from the *code annotation and documentation* section of the survey.

We also asked whether participants had used a set of the coding practices recommended in many of the guidelines. Although just 60 participants reported following specific guidelines, most participants followed at least some recommended practices (Fig 4).

### What are the perceived facilitators to increase reproducible research practices in public health?

Finally, we asked participants to choose their top two of five actions that would facilitate increased use of reproducible research practices. The top selection was *training on reproducible research practices* (n = 109; 50.9%), followed by *requirements by journals to include access to data and statistical code* (n = 95; 44.4%), and 86 (40.2%) chose *requirements by funders to disseminate data and statistical code*. Just 57 (26.6%) and 32 (15%) chose *additional human and financial resources* or *workplace incentives (e*.*g*., *pay increases or credit toward tenure)*.

## Discussion

The crisis in science where error, omission, and fraud are threatening the quality of evidence we rely on has been well-documented [1–3]. While replication is the gold standard for confirming evidence, reproducibility requires fewer resources and increases reliability. Although reproducing research results does not ensure the original analyses were correct or appropriate, the process of reproducing results can both confirm the findings and also uncover questionable and erroneous choices made during data management and analyses [14,15]. Our survey is among the first to examine current practices and challenges to the creation of a culture of reproducibility in public health.

There are several key findings from our survey. First, few participants reported publicly sharing code and data, suggesting that this reproducible research practice is uncommon in public health. The top barrier to data sharing was data privacy, which is often required to obtain human subjects approval from an IRB for gathering health-related data. However, many public health studies use publicly available source data, which would not present privacy concerns for code and data sharing.

In cases where data are not publicly available and cannot be shared due to data use agreements, there are at least three options to promote replication and/or reproducibility. First, it may be possible to de-identify or anonymize data for public sharing within the confines of data use agreements. Second, a simulated data set of the exact structure as the real data set can be created and used to test the statistical code written for the published results, a process known as *quasi-reproducible* research [26,27]. Third, statistical source code can be shared even when data cannot. Sharing well-organized and annotated statistical code allows careful review of data management and analysis procedures. In addition, if complete data collection procedures are reported, sharing statistical code allows researchers to collect new samples and conduct the same analyses. Given that sharing is uncommon and solutions to data privacy concerns exist, training on these options may help to overcome this perceived barrier.

The academic community identified lack of time as another top barrier to using reproducible research practices. While there may be an initial time investment to learn reproducible research practices, adopting recommended strategies and forming habits, such as using descriptive variable names in code, will ultimately result in the reduction of *technical debt*, or the time you cost yourself later by doing things quickly rather than following recommended practices. Of the 60 who used coding guidelines, 50 reported it made life easier, and the guidelines follow principles of reproducibility. There are many other benefits of spending time to properly document and archive your data and statistical code. Shepherd notes some of these immediate benefits including improved record keeping, facilitation of code sharing, and pleasing reviewers [27].

The top facilitators noted were more training, journal requirements, and funding requirements. The identification of training needs is consistent with results of a survey of 190 NIH clinical and basic science researchers [28]. Although participants were reported to have “considered data sharing and reuse important to their work,” most reported a lack of experience of uploading data to a repository [28]. To address research reproducibility training issues, the National Institute of General Medical Sciences (NIGMS) website houses training modules covering a wide range of topics related to research replication and reproducibility including experimental design, statistical considerations, and writing sections of papers; however none of the modules directly addresses practices for sharing statistical code and data at the publication phase of a project. To increase opportunities for continuing education in reproducible research practices, professional organizations and conference organizers might prioritize offering online and in-person workshops on reproducibility.

In addition to training, the identification of requirement by journals and funders as a facilitator to reproducible research is noteworthy. One of the major sources of funding for U.S. public health research is the National Institutes of Health (NIH). The NIH started addressing concerns regarding the rigor of research findings in January 2014, launching several initiatives designed to enhance and encourage reproducible research including an NIH grant policy in 2015 (NOT-OD-16-011) requiring applicants and reviewers to address project reproducibility in applications and reviews. However, there is no guidance on specific details that should be included to ensure statistical code is created and data are shared using reproducible research practices [29]. Although beyond the scope of this discussion, it is worth mentioning that the NIH published a data sharing policy in 2003 [30]. Although numerous repositories are supported by the NIH to facilitate data sharing [31], there is currently no “commons” repository allowing sharing of general public health data [32]. Funding agencies that have not implemented reproducibility requirements could incentivize applicants conducting quantitative work to voluntarily use reproducible practices by adding a score or review incentive (e.g., mulitplier or points) to applications with plans to share code, data, or both.

Journals requiring statistical source code and enough data collection detail (if the data source is not public) to allow users to obtain relevant data could increase the use of reproducible research practices. While some journals suggest making data available on a voluntary basis [33] or even requiring it [34] as a part of the publication process, it is uncommon in our experience as public health researchers. Likewise, in our experience, few journals require use of available reporting guidelines that promote sufficient data collection detail, for example the *STrengthening the Reporting of OBservational studies in Epidemiology* (STROBE) guidelines for observational studies, *Consolidated Standards of Reporting Trials* (CONSORT) for randomized trials, or *Preferred Reporting Items for Systematic Reviews and Meta-Analyses* (PRISMA) for systematic reviews.

One way to encourage journals to adopt reproducibility standards might be to develop a reproducibility rating system for journals. However, adopting reproducibility standards could change what journals need from reviewers or staff and potentially slow the publication timeline [35]. Some examination of which standards are most feasible and effective, and how to efficiently incorporate them into journal processes, would be a worthwhile next step toward this goal. In the meantime, journals without reproducibility requirements could incentivize authors to voluntarily use reproducible practices by adding an indicator (e.g., badge or statement on the title page) on manuscripts with shared code, data, or both. Journal reviewers could also suggest in manuscript reviews that authors adopt reproducible practices such as added detail in methods sections or statistical code sharing.

### Limitations

There are several limitations to our study. This work was exploratory and results may not be generalizable to all public health researchers. Specifically, we surveyed members of APHS, whose members may not represent the entire universe of public health researchers. It is likely that the need for additional training in statistical coding and documentation, as well as data and code sharing, is higher in the general public health community than in our participants. In addition, social desirability may have played a role. For example, 23.3% of our participants reported not including sample sizes in their recent publication; this is at the low end of the range (20% to 80%) of papers with missing or unclear sample size in a study of ten top scientific journals [8]. Despite these limitations, this was the first study we know of to survey public health researchers about use of reproducible practices.

### Recommendations

Although many scientists are aware of the reproducibility problem, solving this problem will require a major cultural shift by the public health community. Our results suggest current professionals, journals, funders, and training programs are all important stakeholders in increasing reproducible research. For *current public health professionals*, we identified important gaps in knowledge and in the use of reproducible research practices, which might be filled by providing accessible training opportunities and guidance. However, old practices die hard and participants reported that *funders* and *journals* requiring reproducible research as a condition of funding and publication could facilitate changes in current practice. Finally, 38 of the 60 participants who used specific guidelines reported they were taught that way, suggesting that incorporating reproducible research into *public health degree programs* may be an effective strategy for increasing use of reproducible practices and improving the quality of public health evidence. Some programs have already introduced this content into the curriculum. To encourage more degree programs to integrate reproducible research practices into the curriculum, the Council on Education for Public Health might consider adding a reproducible research competency to accreditation requirements.
